# Positive roadside edge effects on artificial nest survival in a lowland Atlantic Forest

**DOI:** 10.1002/ece3.5158

**Published:** 2019-06-14

**Authors:** Gleidson Ramos da Silva, Pedro Diniz, Aureo Banhos, Charles Duca

**Affiliations:** ^1^ Programa de Pós‐graduação em Ecologia de Ecossistemas Universidade Vila Velha Vila Velha Brazil; ^2^ Laboratório de Ecologia de Populações e Conservação Universidade Vila Velha Vila Velha Brazil; ^3^ Departamento de Biologia Universidade Federal do Espírito Santo Alegre Brazil

**Keywords:** artificial nest predation, breeding success, edge effect, fragmentation, road ecology

## Abstract

Road construction is considered to be one of the primary causes of forest fragmentation, and little is known about how roads affect bird reproductive success. The objective of this study was to assess the survival rate of artificial nests along an edge associated with a highway and in the interior of a tabuleiro forest. The study was performed at the Sooretama Biological Reserve, on the margins of federal highway BR‐101, between September and October 2015. A total of 168 artificial nests with a Common quail (*Coturnix coturnix*) egg in each nest were placed along six sampling transects, at distances of 2, 25, 50, 100, 200, 400, and 800 m from the highway toward the forest interior. We used logistic regression and estimated daily survival rate (DSR) using the “Nest Survival” function in the program MARK to estimate artificial nest survival and assessed the effect of the distance from the highway. The artificial nest survival rate was significantly higher on the highway margins than at other distances. The results show that artificial nests located up to 25 m from the highway have a greater success probability (over 95%) and a significant decrease in success probability more than 50 m from the highway. Although we cannot rule out other nonroad‐specific edge effects on artificial nest predation, our results suggest that the impacts of the highway (e.g., noise, vibration, visual stimuli) cause predators to avoid the road's surroundings (up to 25 m into the forest) when selecting their feeding sites, which partially supports the predation release hypothesis.

## 1 INTRODUCTION

Road construction is considered to be one of the primary causes of forest fragmentation, resulting in an increase in the amount of edge habitat (Coffin, 2007; Fearnside, 1990; Laurance, Goosem, & Laurance, 2009). Edges created by roads are more abrupt than the edges common in many natural landscapes, which are more diffuse, thus increasing problems associated with an edge effect (Benítez‐López, Alkemade, & Verweij, 2010; Forman & Deblinger, 2000). The relationship between roads and biodiversity involves many variables, including environmental, social, cultural, and economic factors, which are intertwined in an interaction network and reflect the developmental history of a country or region (Freitas, Hawbaker, & Metzger, 2010).

The barriers formed by roads not only fragment the landscape but also interrupt the flow of some species and lead to changes in the ecological relations among the edge‐using species (De Oliveira, Alberts, & Francisco, 2011; Develey & Stouffer, 2001; Forman & Alexander, 1998 ). Fauna is also disturbed by the noise pollution originating from roads. There is evidence that the noise caused by vehicle transit is an important factor affecting bird communities near roads (Halfwerk, Holleman, Lessells, & Slabbekoorn, 2011; Reijnen, Foppen, TerBraak, & Thissen, 1995; Ware, McClure, Carlisle, & Barber, 2015). Finally, collisions with vehicles are another important feature shaping animal populations near roads (Trombulak & Frissel, 2000).

Many studies have already assessed the edge effect on bird reproduction (reviews in Lahti, 2001; Batáry & Báldi, 2004; Vetter, Rücker, & Storch, 2013), but most of these studies have been performed at forest edges associated with pastures or other vegetation structures. Several studies have argued that nest predation be elevated near habitat edges (reviewed by Chalfoun, Thompson, & Ratnaswamy, 2002). Indeed, some studies have shown that the predation rate of natural and artificial nests is higher closer to edges (Arbeiter & Franke, 2018; Askins, 1995; Cox, Thompson, & Faaborg, 2012; Marini, Robison, & Heske, 1995). However, studies have shown contrasting results for the relationship between forest fragment size and the predation rates of artificial nests as well as a lack of any increase in nest predation rates at fragment edges compared to the forest interior (Duca, Gonçalves, & Marini, 2001; França & Marini, 2009; Luo, Zhao, Ma, Li, & Xu, 2017; Watson, Whittaker, & Dawson, 2004).

A recent meta‐analytical study found no edge effect on nest predation across tropical species, but a higher probability of nest predation along forest edges when pooling together data from tropical and temperate species (Vetter et al., 2013). In addition, this study showed a high heterogeneity in the effect of roadside edges on nest predation across studies (Vetter et al., 2013). There have been few experimental studies on the factors affecting nest predation intensity near habitat edges in tropical forests (Coelho, 1999; Marini et al., 1995), especially regarding forest edges associated with roads (Batáry & Báldi, 2004; Vetter et al., 2013). Therefore, it is important to perform more studies to assess the influence of fragmentation processes and edge effects related to different types of adjacent land uses (Vetter et al., 2013), to understand the effects of edges associated with roads.

Several mechanisms have been proposed to explain why nest predation may increase or decrease near the edge. Nest predation should decrease with the increasing distance from the edge, and be higher in small fragments, in circumstances where the predators choose small patches and edges for foraging (Chalfoun et al., 2002; Gates & Gysel, 1978; Wilcove, Mclellan, & Dobson, 1986). On the other hand, nest predation can be lower near edges whether nest predators are less dense or less active near the edges due the avoidance of unsuitable habitats (Khamcha et al., 2018) or whether the predators have lower foraging efficiency near the edges due to edge impacts on vegetation structure (Harper et al., 2015). The species‐specific responses of predators to edges may determine the overall effect of edge on nest predation (Khamcha et al., 2018).

The predation release hypothesis states that prey are favoured near roads because roads negatively affect the predators' abundance and/or foraging activity due to road mortality and traffic disturbance (Downing, Rytwinski, & Fahrig, 2015; Fahrig & Rytwinski, 2009; Rytwinski & Fahrig, 2013). Although it is well known the negative impacts of roads on bird predators (Trombulak & Frissel, 2000; Zabala et al., 2006), studies failed to support the prediction of lower adult and nest predation near roads (DeGregorio, Weatherhead, & Sperry, 2014; Dziadzio, Smith, Chandler, & Castleberry, 2016), probably because some predators can be favoured by roads (DeGregorio et al., 2014).

In this study, we assessed the survival probability of artificial, open cup nests along edge areas associated with a highway and in the interior of an Atlantic forest. We tested the hypothesis that roadside edges will have an (positive or negative) impact on nest survival, by evaluating the prediction that artificial nest survival will be higher or lower near roadside edges than in the interior of the forest. We discussed the possible mechanisms underlying the edge‐interior gradient in nest predation. We argue our study contributes to understand the spatial diversity of edge effects on nest survival in the tropics.

## 2 MATERIAL AND METHODS

### 2.1 Study site

**Figure 1:**
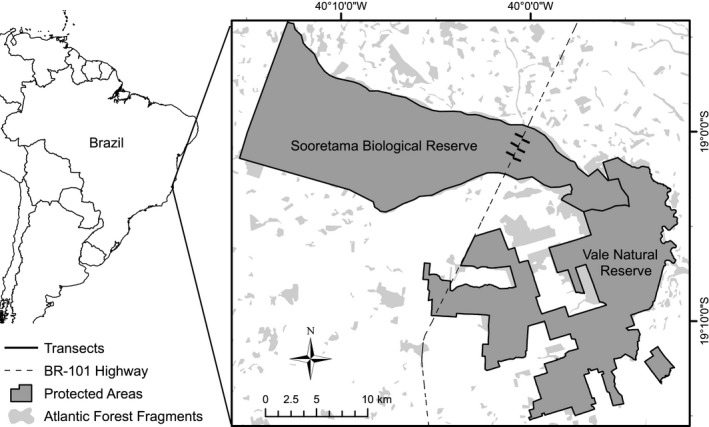
Map of South America and Brazil, with location of the Sooretama Biological Reserve with the BR‐101 highway and transects used in the artificial nest experiment

The experiment was performed at the Sooretama Biological Reserve (hereafter SBR), located in the Sooretama, Linhares, Vila Valério and Jaguaré municipalities, in northern Espírito Santo state, Brazil, between 18º53′ and 19º05′S and 39º55′ and 40º15′W (Figure 1). The SBR is considered one of the largest “tabuleiro” forest (dense lowland rainforest located on flat terrain) remnants in southeast Brazil and has a forested area of 24,250 ha (Peixoto et al., 2008; Peixoto & Simonelli, 2007). The SBR is located in the *barreiras* formation, characterized by a wide relief, with low hills of the “tabuleiro” type and a maximum altitude of 200 m above sea level (Paula & Soares, 2011) located in the Atlantic Forest biome. According to the Köppen classification, the climate in the region is tropical with a dry winter and rainy summer (Aw) (Alvares, Stape, Sentelhas, Gonçalves, & Sparovek, 2013). The cumulated precipitation is 1,403 mm, and the average annual temperature is 23.6ºC (Alvares et al., 2013; Magnago, Rocha, Meyer, Martins, & Meira‐Neto, 2015).

According to the phytogeographic system established by the Brazilian Institute of Geography and Statistics (Instituto Brasileiro de Geografia e Estatística—IBGE), the primary vegetation formation found in the region is Lowland Dense Ombrophilous Forest (Tabuleiro Forest), a forest comprised by a coastal stretch of flatland forests on the geologic formation of the same name (Coastal Tabuleiro) (IBDF & FBCN, 1981).

### 2.2 Artificial nests experiment

The experiment was conducted from 22 September 2015 to 07 October 2015, at the start of the reproductive season of most birds in the region (Sick, 2001). The experiment was conducted along the margins of the federal highway BR‐101 (Mario Covas highway), which passes transversally through the reserve for approximately 5.3 km (Figure 1). This is a paved, two‐lane highway with 15 m wide and 60 km/hr of speed limit at the section that passes through the reserve. Traffic on this highway includes all categories of vehicles, and traffic flow is considered heavy, approximately 8,000 vehicles per day, though there is not quantitative information available.

Six transects were placed perpendicular to the highway, with sampling points at 2, 25, 50, 100, 200, 400, and 800 m from the highway toward the forest interior. Four nests were positioned at a height of approximately 1.5 m from the ground at each sampling point, parallel to the road, spaced 25 m apart, resulting in 24 nests per distance from the highway, and an overall total of 168 nests.

The nests were constructed from grass bundles arranged in spirals and sewn together to keep them from falling apart. One common quail (*Coturnix coturnix*) egg was placed in each nest, and the nests were exposed to predators for 15 days, which is the average incubation time of Passeriformes in the region (Marques‐Santos, Braga, Wischhoff, & Roper, 2015; Sick, 2001). The nests were monitored, and their content was assessed (predated or intact) every 3 days. The nests were considered as predated when the eggs had been damaged or removed.

### 2.3 Data analysis

The apparent predation rate was calculated as the percentage of predated nests by dividing the total number of predated nests at each distance by the total number of nests at each distance, resulting in the success percentage.

Variation in nest survival among categories of distances from the highway was analyzed with three approaches: (a) modeling and (b) comparison of daily survival rate (DSR), and (c) logistic regression. First, daily survival rate (DSR), the probability that a nest survives 1 day within a specific time interval (Dinsmore, White, & Knoff, 2002), were calculated using the “Nest Survival” function in Program MARK (Cooch & White, 2012). This function allows for the development and comparison of models of daily survival containing different temporal and spatial covariate effects.

The nest survival model needs a minimum of four pieces of information to estimate DSR, namely the first day encountering the nest, the last day the nest was checked and not depredated, the last day the nest was checked, and the fate of the nest: depredated or not. Because we used artificial nests, the first day encountering the nest was always the first experimental day. The record of each nest consistently lasted from day 1 (first experimental day) to day 16 (last experimental day), and then, the maximum duration of egg exposure to predation was 15 days. Each set of 24 nests at the distances from the highway (at 2, 25, 50, 100, 200, 400, and 800 m from the highway) was considered a group, and then, there were seven groups. Therefore, groups represent the distance from the highway (road effect).

To test hypotheses, we constructed generalized linear models (Program MARK) to evaluate the daily survival of artificial nests at each distance from the highway (at 2, 25, 50, 100, 200, 400, and 800 m). We ranked the models based on Akaike's information criterion (AIC) values, where models with ΔAIC ≤2 were considered to have similar ability to explain variation in the data set (Burnham & Anderson, 2002). We constructed models considering time (*t*), group (*g*), and combination among them (g*t). In the models, groups represent distances from the highway. We also constructed models grouping the groups in different ways to assess the road effect on DSRs. For instance, a model with group 1 isolated from the others (*g*1), groups 1 and 2 as only one group (*g*1–2), groups 1 and 2 as only one group and groups 3–7 as another group (*g*1–2, *g*3–7), group 3–7 as only one group (*g*3–7). See Table 1 for other models.

**Table 1:** Model selection of nest survival (*S*) based on Akaike's information criteria (AIC)

Models	AICc	ΔAICc	wi	*K*	Deviance
*S*_(_ *_g_*_1)_	305.99	0.00	0.33	2	301.99
*S*_(_ *_g_*_3−7)_	306.32	0.32	0.28	3	300.30
*S*_(g1−2, _ *_g_*_3−7)_	306.56	0.57	0.25	2	302.56
*S*_(_ *_g_*_3)_	309.99	4.00	0.05	2	305.98
*S*_(_ *_t_*_)_	310.62	4.62	0.03	6	298.58
*S*_(_ *_g_*_)_	311.64	5.65	0.02	7	297.59
*S*_(_ *_g_*_1−2)_	311.88	5.89	0.02	6	299.84
*S*_(.)_	312.21	6.22	0.02	1	310.21
*S*_(_ *_g_*_2)_	313.42	7.43	0.01	2	309.41
S_(_ _g__*_ *_t_*_)_	391.41	85.42	0.00	65	257.22

Note

The model notion (*g*) is a group effect indicating that survival was estimated separately for each distance from the highway. (.) Indicates constant survival, and (*t*) a time effect; numbers indicated different groups that represent distances from the highway. Total AIC, difference of AIC of each model relative to the top model (ΔAIC), Akaike's model weight (wi), numbers of parameters (*K*), and deviance are provided for each model.

Nest success estimates were also compared between the different distances with Mayfield's protocol (Hensler & Nichols, 1981; Mayfield, 1975 ), which was also used to calculate nest survival rate based on exposure time. In this analysis, differences in survival probability among the different distances from the highway were evaluated pairwise using the *Z*‐test adapted to Mayfield's protocol according to Hensler and Nichols (1981).

Finally, we performed logistic regression in R (*glm* function, R Core Team, 2017) to evaluate how nest survival (0: successful, 1: depredated) varies with distance from the highway (a factor with seven distance levels). Nest exposure time (in days) was included as an offset in this model. We compared the models with and without (i.e., null model) distance variable with likelihood ratio test. If we found an effect of distance to the highway upon the probability of the nest being depredated, we carried out post hoc comparisons among levels of distance using the packages *multcomp* (Hothorn, Bretz, & Westfall, 2008) and *lsmeans* (Lenth, 2016). *p* values in multiple comparisons were controlled for false discovery rates (Benjamini & Hochberg, 1995). Because we have seven levels of distance, we considered significant *p* values those lowered than 0.1 in post hoc comparisons.

## 3 RESULTS

**Figure 2:**
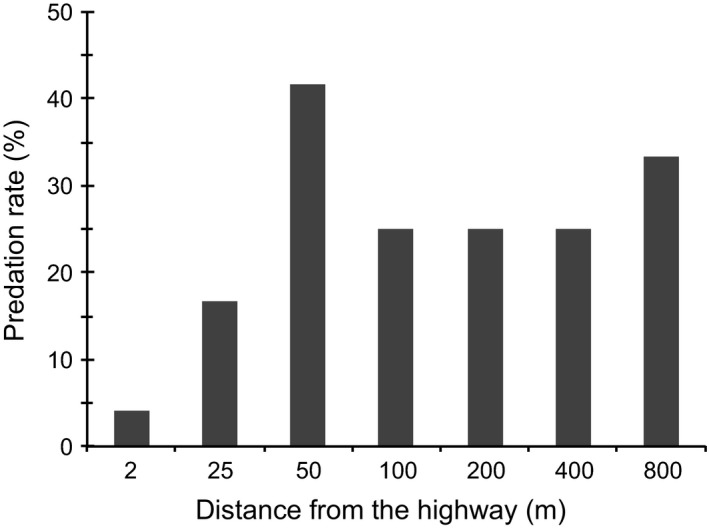
Predation rate (%) of artificial nests in the Sooretama Biological Reserve, Brazil

Of the 168 nests used in the experiment, 24.4% were depredated after 15 days of exposure. The apparent predation rate of nests two meters from the highway was 4% and increased starting at 25 m (16%), with the largest predation rate observed at 50 m (41%) (Figure 2).

The constant generalized linear model [*S*_(.)_] and model indicating time effect on DSR [*S*_(_ *_t_*_)_] had little support (ΔAIC > 2). In contrast, models describing differences from groups 1 and 2 (distances 2 and 25 m from the highway) to others had greater support, with the models *S*_(_ *_g_*_1)_, *S*_(_ *_g_*_3–7)_, and *S*_(1–2,_ *_g_*_3–7)_ comprising 86.2% of the weight of evidence (Table 1).

**Figure 3:**
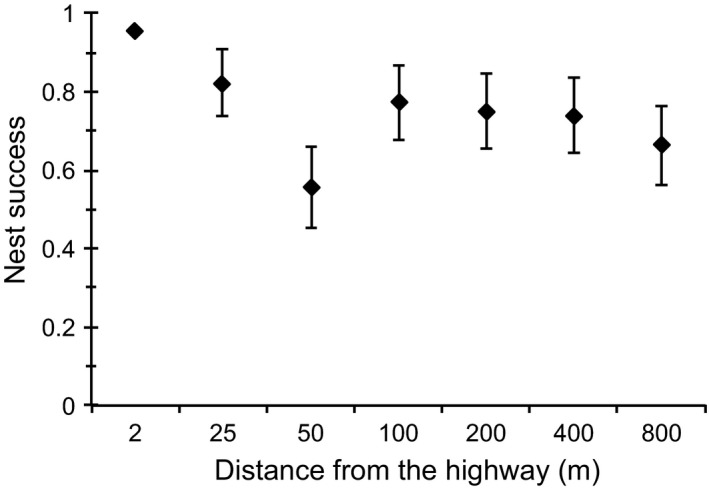
Nest success (±*SD*) of artificial nests in relation to their distance (m) from highway BR‐101 in the Sooretama Biological Reserve, Brazil

The probability of success for nests located between two and 25 m from the highway varied from 82% to 96%, whereas that of nests located 50 m or more from the highway varied from 58% to 77% (Table 2). Survival rates of nests located up to 25 m from the highway were significantly different from the other distances, with the greatest success probabilities observed for nests located between 2 and 25 m from the highway (Table 3, Figure 3).

**Table 2:** Daily survival rate, standard error, confidence interval, and apparent nest success of groups (distance from the highway) estimated by the group effect model [*S*_(_ *_g_*_)_] for artificial nests experiment in the Sooretama Biological Reserve, Brazil

Distance from highway (m)	Daily survival rate	Standard error	95% confidence interval	Nest success
2	0.997	0.003	0.980–0.999	0.956
25	0.987	0.006	0.967–0.995	0.822
50	0.964	0.011	0.935–0.981	0.577
100	0.983	0.008	0.960–0.993	0.773
200	0.981	0.008	0.957–0.991	0.750
400	0.980	0.008	0.957–0.991	0.739
800	0.973	0.009	0.947–0.987	0.663

**Table 3:** *Z*‐test values comparing the nest survival probabilities among the distances from the highway in the Sooretama Biological Reserve, Brazil

Distance from highway (m)	25	50	100	200	400	800
2	*z* = 1.48 *p* = 0.14	*z* = 3.54 ***p* ˂ 0.01**	*z* = 2.19 ***p* = 0.03**	*z* = 2.17 ***p* = 0.03**	*z* = 2.18 ***p* = 0.03**	*z* = 2.83 **p ˂ 0.01**
25		*z* = 1.95 ***p* = 0.05**	*z* = 0.72 *p* = 0.47	*z* = 0.69 *p* = 0.49	*z* = 0.70 *p* = 0.48	*z* = 1.31 *p* = 0.19
50			*z* = 1.20 *p* = 0.23	*z* = 1.24 *p* = 0.22	*z* = 1.22 *p* = 0.22	*z* = 0.61 *p* = 0.54
100				*z* = 0.03 *p* = 0.97	*z* = 0.02 *p* = 0.99	*z* = 0.58 *p* = 0.56
200					*z* = 0.02 *p* = 0.99	*z* = 0.62 *p* = 0.54
400						*z* = 0.60 *p* = 0.55

Bold values indicate significant results for Z‐tests.

Logistic regression also demonstrated an overall effect of distance from the highway upon the probability of nest being depredated (likelihood ratio test, *χ* ^2^ = 21.48, *df* = 6, *p* = 0.002). Probability of the nest being depredated was lower at the margin (2 m) of the highway than from 50 up to 800 m toward the interior (post hoc comparisons, *p* < 0.1), whereas nest was more likely to be depredated at 50 m in comparison with all the other distance levels (post hoc comparisons, *p* < 0.1), except with 800 m distant from the highway. Finally, the probability of nest depredation was higher at 25 m than at 50 m from the highway (*p* = 0.05).

## 4 DISCUSSION

Our results support the hypothesis that roadside edge impacts artificial nest survival (Khamcha et al., 2018). Although there was a slight trend toward an increasing predation rate with increasing distance from the highway, our results indicate that predation of artificial nests increases with distance from the highway only in the first 50 m, with no apparent increase in the other distances. In other words, both the predation and artificial nest success probability exhibited differences between the edge and interior and a considerable roadside edge effect existed up to a distance of 25–50 m. A similar pattern of low nest predation up to 25 m from the road was found in a road with high traffic density bordering woodlands in the Iberian Peninsula (Pescador & Peris, 2007). This result was less noticeably near the roads with medium and low traffic density (Pescador & Peris, 2007) or unpaved roads (DeGregorio et al., 2014), indicating that traffic density in paved roads can increase the positive roadside edge effect on nest predation.

Why was artificial nest predation lower near the roadside edge in this tabuleiro forest? Two hypotheses could explain this pattern: predation release hypothesis and nonroad‐specific edge effects hypothesis. Our results confirmed a key prediction of predation release hypothesis that suggests a decrease in nest predation attempts near the roads (DeGregorio et al., 2014; Dziadzio et al., 2016; Fahrig & Rytwinski, 2009). Therefore, road mortality or mortality risk, and/or road disturbance (e.g., noise) may be negatively affecting predator abundance near the road (Downing et al., 2015; Fahrig & Rytwinski, 2009; Rytwinski & Fahrig, 2013). Some studies in open habitat types have shown that the densities of many species decrease next to roads (Benítez‐López et al., 2010; Clark & Karr, 1979; Fahrig & Rytwinski, 2009; Illner, 1992), which can be a response to noise (Pescador & Peris, 2007). In addition, road avoidance by forest birds and mammals can extend hundreds of meters from busy roads (Benítez‐López et al., 2010; Forman & Deblinger, 2000) and bird and mammals species are important nest predators in the neotropics (Menezes & Marini, 2017). Our results, however, suggest an edge‐interior space use gradient by predators that extend at least 25 m into the forest. Therefore, our results support the idea that roads negatively affect predators.

However, the predation release hypothesis also predicts that prey are more abundant near the roads (Downing et al., 2015; Fahrig & Rytwinski, 2009; Rytwinski & Fahrig, 2013). A previous study conducted at the same edge‐interior gradient at Sooretama Biological Reserve found no evidence of edge effects on abundance of bird species (Silva, 2015). Therefore, our results only partially support the predation release hypothesis, suggesting that predation release advantage for birds may be not compensating negative effects of the road (road disturbance and mortality or reduced habitat quality). Although road edges can be a safe place against predators, other factors not evaluated in the current study may negatively affect the reproduction of birds near the highways. For example, traffic noise can make marginal habitats degraded for reproduction (Halfwerk et al., 2011; Ware et al., 2015).

Alternatively, we can explain the low artificial nest predation at the forest edge with others factors that are not road‐specific. There is strong evidence of edge effects on vegetation structure (e.g., reduced tree density and canopy close to the forest edge) and microclimate (e.g., higher temperature, stronger winds, and low air humidity at the forest edge than at the interior) (Harper et al., 2015; Kunert, Aparecido, Higuchi, Santos, & Trumbore, 2015; Magnago et al., 2015). Changes in vegetation structure can in turn alter habitat selection (Pasinelli, Grendelmeier, Gerber, & Arlettaz, 2016; Wolfe, Johnson, & Ralph, 2014) and/or foraging efficiency (Schneider, Low, Arlt, & Part, 2012) by predators, for example, nest detection by predators may be reduced at the forest edges (Martin & Roper, 1988; Picman, 1988). Accordingly, nest predation risk can be associated with vegetation structure or landscape features (Díaz & Carrascal, 2006; Seibold et al., 2013). Therefore, further studies are needed to disentangle the roadside‐specific edge effects on nest predation from other edge effects not necessarily to roads.

Although the short distance up to which artificial success probability was affected by the highway (of 25 up to 50 m) may seem of little importance in terms of impacts, in regard to linear structures such as roads, this effect must be considered on a scale of hundreds to thousands of square kilometers where this road is located. These effects are therefore important for sites whose goals include preservation and the avoidance of impacts and for protected areas crossed by roads that have some of their protection functions disrupted by road impacts, as in the case of SBR. Our results are particularly important because SBR is the largest continuous area of tabuleiro forest in the Atlantic forest, and recognized by its species richness, trophic complexity, and refuge for threatened species (reviewed in Magnago et al., 2015). We encourage further studies to address the effect of road with different widths and traffic noise on nest survival. This would help road ecologists to better predict road‐upgrading impacts on nest survival.

We conclude that the highway BR‐101 affects the success probability of artificial nests within a tabuleiro, Atlantic forest. We suggest that species reproducing between two and 25 m from the highway may attain greater reproductive success. We partially support the predation release hypothesis, which predicts that the impacts of the highway (e.g., noise, vibration, visual stimuli) cause predators to avoid the road's surroundings when selecting their feeding sites.

## CONFLICT OF INTEREST

None declared.

## AUTHOR CONTRIBUTIONS

GRS conceived the ideas and collected the data; AB and CD conceived the ideas and designed experiment; GRS, PD, and CD analyzed the data and led the writing of the manuscript. All authors contributed critically to the drafts and gave final approval for publication.

## Data Availability

Data associated with this manuscript will be uploaded to Dryad Digital Repository.
